# Curcumin inhibits the TGF-β1-dependent differentiation of lung fibroblasts via PPARγ-driven upregulation of cathepsins B and L

**DOI:** 10.1038/s41598-018-36858-3

**Published:** 2019-01-24

**Authors:** Ahlame Saidi, Mariana Kasabova, Lise Vanderlynden, Mylène Wartenberg, Ghania Hounana Kara-Ali, Daniel Marc, Fabien Lecaille, Gilles Lalmanach

**Affiliations:** 1 Université de Tours, Tours, France; 2 INSERM, UMR 1100, Centre d’Etude des Pathologies Respiratoires (CEPR), Equipe «Mécanismes Protéolytiques dans l’Inflammation», Tours, France; 3 INRA, UMR 1282, Infectiologie et Santé publique (ISP), Centre INRA Val de Loire, Nouzilly, France; 4 Present Address: Pharmaceutical Product Development, Sofia, Bulgaria

## Abstract

Pulmonary fibrosis is a progressive disease characterized by a widespread accumulation of myofibroblasts and extracellular matrix components. Growing evidences support that cysteine cathepsins, embracing cathepsin B (CatB) that affects TGF-β1-driven Smad pathway, along with their extracellular inhibitor cystatin C, participate in myofibrogenesis. Here we established that curcumin, a potent antifibrotic drug used in traditional Asian medicine, impaired the expression of both α-smooth muscle actin and mature TGF-β1 and inhibited the differentiation of human lung fibroblasts (CCD-19Lu cells). Curcumin induced a compelling upregulation of CatB and CatL. Conversely cystatin C was downregulated, which allowed the recovery of the peptidase activity of secreted cathepsins and the restoration of the proteolytic balance. Consistently, the amount of both insoluble and soluble type I collagen decreased, reaching levels similar to those observed for undifferentiated fibroblasts. The signaling pathways activated by curcumin were further examined. Curcumin triggered the expression of nuclear peroxisome proliferator-activated receptor γ (PPARγ). Contrariwise PPARγ inhibition, either by an antagonist (2-chloro-5-nitro-N-4-pyridinyl-benzamide) or by RNA silencing, restored TGF-β1-driven differentiation of curcumin-treated CCD-19Lu cells. PPARγ response element (PPRE)-like sequences were identified in the promoter regions of both CatB and CatL. Finally, we established that the transcriptional induction of CatB and CatL depends on the binding of PPARγ to PPRE sequences as a PPARγ/Retinoid X Receptor-α heterodimer.

## Introduction

Pulmonary fibrosis (PF), a distinctive form of chronic interstitial pneumonia^1–4^, is a progressive and irreversible fibrotic disease. The early phase of PF corresponds to an ongoing alveoli injury, followed by infiltration of inflammatory cells as well as by an abnormal activation and proliferation of extracellular matrix (ECM)-producing cells (fibroblasts, myofibroblasts). The ensuing overproduction of ECM components results in a progressive scarring, an aberrant repair and the destruction of alveolar architecture, which lead to an irreversible decline in pulmonary function (average survival of 3–5 years after diagnosis)^1^. Fibroblastic foci show an enhanced activation response to profibrogenic cytokines. Among them, transforming growth factor-β1 (TGF-β1) that is a key mediator in PF^5^ stimulates both the proliferation of lung fibroblasts and their subsequent differentiation into myofibroblasts^6–8^. Unlike fibroblasts, myofibroblasts overproduce alpha-smooth muscle actin (α-SMA), a testimonial profibrotic biomarker, and type I collagen. As an outcome, the dysregulated ECM remodeling/turnover is closely associated with molecular mechanisms that result from an alteration of the proteases/antiproteases balance^9^. The consequences of this compromised homeostatic balance and its contribution to PF pathogenesis were recently reviewed by Crestani and coworkers^10^. Besides metalloproteinases, serine and cysteine proteases (cathepsins and calpains), along with their cognate inhibitors, participate in signaling pathways and proteolytic mechanisms associated with fibrogenesis^11–13^.

Human lysosomal cysteine cathepsins comprise 11 members (cathepsins B, C, F, H, K, L, O, S, V, W and X)^14,15^. Cathepsins have long been regarded as ubiquitous ancillary enzymes, predominantly involved in the recycling and degradation of proteins. However they also contribute in numerous specific physiological processes, such as the presentation of antigenic peptides and the maturation of thyroid hormones or neuropeptides^16,17^. Besides the unique and well-documented collagenolytic property of cathepsin K (CatK)^18,19^, other cathepsins (i.e. B, L, S, and V), which also exhibit collagenolytic and elastinolytic activities, are essential players in the tissue remodeling of ECM and basement membrane (BM) as well as in the shedding of adhesion molecules and extracellular receptors^20–22^. It was recently established that cathepsins play distinctive roles in lung homeostasis and pathophysiological events (e.g. asthma, emphysema). Besides their primary location in acidic compartments, secreted cathepsins were also found in bronchoalveolar lavage fluids (BALFs) from patients suffering from pulmonary disorders (e.g. sarcoidosis, silicosis)^23,24^.

Recent studies have uncovered diverse roles for cathepsins during fibrotic processes. Lung fibroblasts derived from CatK-deficient mice showed a decreased collagenolytic activity, while increased levels of CatK protected lungs from bleomycin-induced fibrosis by reducing ECM deposition^25,26^. CatL from heart fibroblasts participates in collagen turnover and may be cardioprotective during myocardial fibrosis^27^. Otherwise, CatL and CatB, which are overexpressed in steatosis and hepatic fibrosis, may serve as diagnostic markers for chronic liver diseases^28^. The limited availability of human primary fibroblasts (i.e. clinical samples obtained by lung biopsy) prompted us to develop a relevant model of differentiated lung fibroblasts (CCD-19Lu cells) to decode the role of cathepsins in fibrotic processes. Inhibition of CatB impaired α-SMA expression, and we demonstrated that the activation of TGF-β1, which in turn triggers the canonical Smad signaling pathway, partly depends on CatB activity. Moreover, we established that TGF-β1 drives cystatin C oversecretion during myodifferentiation, thereby inhibiting the collagenolytic activities of extracellular cathepsins^29^. Accordingly we observed a significant increase of cystatin C in BALFs from patients suffering from idiopathic pulmonary fibrosis, suggesting its possible use as a clinical marker of fibrosis^30^. These results also reflect the dysregulation of active cathepsins in lung, which in turn can promote myofibrogenesis.

Despite numerous studies and clinical trials, current treatments of fibrosis still suffer from their modest effectiveness^31–34^. Curcumin (a.k.a. diferuloylmethane) is the major component of turmeric powder extracted from Curcuma longa. Besides its use as a food flavoring and coloring spice^35^, curcumin is used as antioxidant and anti-inflammatory drug in Chinese medicine since hundreds of years^36^. This polyphenol exhibits antiviral, antibacterial, antifungal and antitumoral activities^37^. Also, curcumin inhibits proliferation and activation of hepatic stellate cells during liver fibrosis^38^, and compromised extracellular-signal-regulated kinase (ERK) phosphorylation^39^. Some years ago, Brömme and coworkers observed that curcumin increases the expression of lung CatK and prevented collagen accumulation in a murine model of bleomycin-induced pulmonary fibrosis^26^. According to these seminal results, the goal of the present study was to decipher the molecular mechanisms underlying the antifibrotic properties of curcumin, using human lung CCD-19Lu fibroblasts.

## Results and Discussion

### Curcumin inhibits the TGF-β1-induced differentiation of human lung fibroblasts

**Figure 1:**
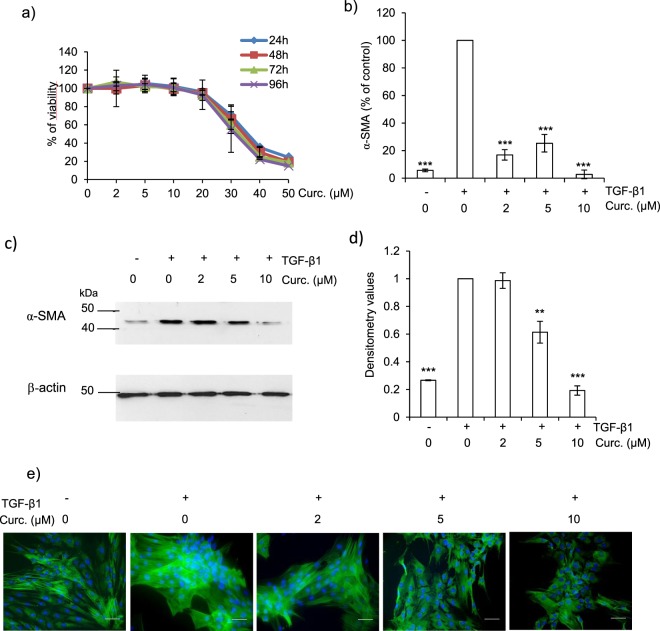
Effect of curcumin on α-SMA expression of human CCD-19Lu myofibroblasts. (**a**) Three days after induction of the differentiation of CDD-19Lu cells into myofibroblasts by TGF-β1 (5 ng/ml), curcumin (0–50 µM) was added for different time intervals (24–96 h). The cell viability was then determined by MTS assay. Results (average values) were normalized using untreated cells as control (100% of viability) (n = 3). (**b**) Three days after addition of TGF-β1, myofibroblasts were treated with curcumin (0–10 µM) for 48 h. Analysis of α-SMA expression was performed by quantitative real time PCR analysis of α-SMA transcripts. Data are expressed as percentage relative to untreated control. (**c**) In parallel, myofibroblasts layers were harvested and lysed. Samples were submitted to electrophoresis (12% SDS-PAGE, under reducing conditions) before the protein level of α-SMA was analyzed by western blot using a mouse α-SMA antibody. β-actin was used for load control. A representative sample of three independent experiments is shown. Full-length blots are presented in Supplementary Fig. 5. (**d**) Corresponding WB densitometric analysis of the expression level of α-SMA; normalized data correspond to the average of three independent experiments, using β-actin as control. (**e**) Cells were treated with curcumin for 48 h as reported above. The green staining corresponds to the immunolabeling of α-SMA. Nuclei were stained by DAPI (blue). Scale bar represents 100 µm.

**Figure 2:**
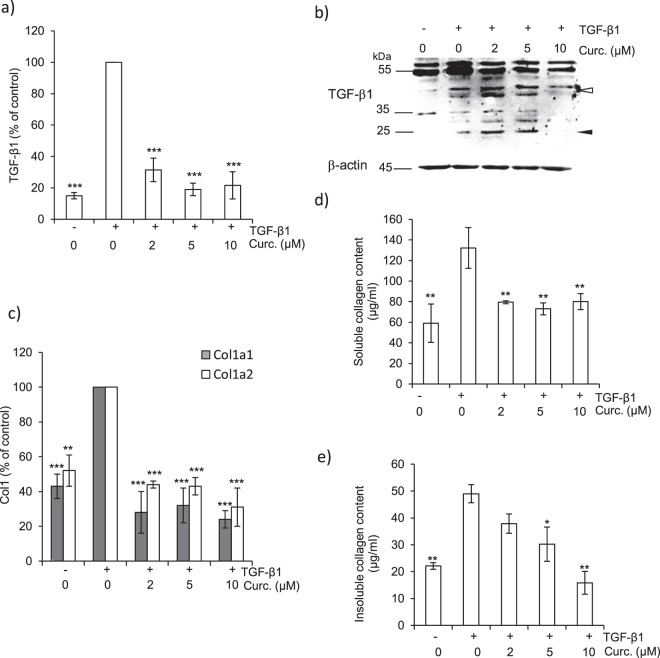
Consequences of curcumin treatment on profibrotic markers of lung myofibroblasts. (**a**) After treatment of CCD-19Lu cells with curcumin (0–10 µM) for 48 h, TGF-β1 transcripts were analyzed by quantitative real time PCR. Normalized data are expressed as percentage relative to untreated control (n = 3). (**b**) Western blot analysis of TGF-β1 expression level. A representative sample of three independent experiments is shown. White arrows indicate precursor pro-region forms; black arrows indicate dimer forms of TGF-β1. β-actin was used for load control. Full-length blots are presented in Supplementary Fig. 5. (**c**) Transcriptional analysis of Col1a1 (collagen type I, alpha-1; white bar) and Col1a2 (collagen type I, alpha-2; grey bar) mRNA by quantitative real time PCR analysis. Normalized data are expressed as percentage relative to untreated control (n = 3). (**d**) Dosage of soluble collagen in culture media (Sircol assay) (n = 3). (**e**) Dosage of insoluble collagen in cell lysates (Sircol assay) (n = 3).

Given both the scarcity of human clinical specimens (e.g. lung IPF biopsies) and the discordance in the expression of lung cathepsins and their endogenous inhibitors between murine bleomycin-induced fibrosis and human pulmonary fibrosis^40^, human CCD-19Lu fibroblasts appeared to be a pertinent model to decipher molecular mechanisms and signaling pathways associated with human pulmonary myofibrogenesis^29^. A hallmark in the development of fibrosis is the TGF-β1-dependent activation, proliferation and differentiation of fibroblasts to α-SMA-expressing myofibroblasts that secrete excessive amounts of collagen. First, we analyzed the cell viability of TGF-β1-differentiated CCD-19Lu fibroblasts, following a curative treatment by curcumin (0–50 µM) for different times (24–96 h). Curcumin inhibited cell growth in a dose-dependent manner (Fig. 1a) at concentrations greater than 20 µM (IC_50_ = 35 μM) after one-day exposure. We therefore chose a concentration range of 0–10 µM for all subsequent experiments since no cell toxicity was measured. Fibroblasts stimulated with TGF-β1 (5 ng/ml) were further treated with curcumin (0–10 µM) during 48 h, then total RNA was isolated and α-SMA expression level determined by real-time quantitative PCR (Fig. 1b). The expression of α-SMA mRNA in curcumin-treated (10 µM) myofibroblasts was reduced to less than 5% relative to untreated control. This was confirmed by an immunoblot analysis of the protein level of α-SMA (Fig. 1c,d) as well as by immunofluorescence (Fig. 1e). The level of α-SMA in curcumin-treated myofibroblasts was similar to that observed for undifferentiated CCD-19Lu fibroblasts. We reported previously a significant and similar reduction of α-SMA expression in the presence of CA-074Me (L-3-trans-(propylcarbamyl)oxirane-2-carbonyl)-L-isoleucyl-L-proline methyl ester), a cell permeable pharmacological inhibitor of CatB or after CatB silencing by a specific siRNA^29^, raising the question of a possible relationship between the antifibrotic effects of curcumin and the profibrotic activity of CatB. Since TGF-β1 regulates ECM (including type I collagen) deposition in the fibrotic tissue^37^, we analyzed in a second step the expression of both endogenous TGF-β1 and collagen following addition of curcumin. Curcumin significantly decreased the expression of TGF-β1 mRNA, down to levels similar to those observed in undifferentiated fibroblasts (Fig. 2a). Subsequently we observed reduced amounts of both 50-kDa TGF-β1 (proform) and its 25-kDa bioactive form (mature dimer) in the presence of curcumin (10 µM) (Fig. 2b). Additional immunoreactive bands corresponding to variant glycosylated forms were observed as reported previously (confirmed by treatment with endo-β-N-acetylglucosaminidase F1)^41^. Of note, we previously observed an opposite effect after the pharmacological inhibition of CatB or after its specific silencing, with increased amounts of the 50-kDa proform of TGF-β1 and impaired release of its 25-kDa form^29^. Taken together, our data likely support a cellular crosstalk between TGF-β1, CatB and curcumin. Otherwise, it is well established that the two genes encoding the α-1 and α-2 chains of type I collagen (Col1a1 and Col1a2) are upregulated during fibrotic processes (for reviews^42–44^). Transcriptional analysis of both Col1a1 and Col1a2 by qRT-PCR showed that their mRNA expression is decreased by more than 50% in curcumin-treated cells (Fig. 2c). Measurement of fibrillar collagen in conditioned media confirmed that curcumin significantly reduced the amounts of soluble collagen (Fig. 2d). Similarly, we observed a significant decrease of insoluble cell layer-associated collagen (Fig. 2e). Following curcumin treatment, the levels of both soluble and insoluble collagen were similar to their observed constitutive level for undifferentiated fibroblasts, attesting that curcumin down-regulated both TGF-β1 and collagen I expression and thus could impair or delay myofibrogenesis. In agreement with these results, it has been shown that curcumin inhibits both myofibrogenesis and collagen secretion in mice following bleomycin-induced lung injury^39,45^. Likewise, curcumin attenuated type I collagen production in an experimental pulmonary fibrosis in rats and showed beneficial antifibrotic effects in a rodent model of obstructive nephropathy^46,47^. Of note, a similar decrease of soluble and insoluble type I collagen that was previously observed in TGF-β1-differentiated lung fibroblasts after treatment by the cathepsin inhibitors E-64d (i.e. (2S, 3S)-trans-epoxysuccinyl-L-leucylamido-3-methylbutane ethyl ester) or CA-074Me^29^. This prompted us to evaluate the effect of curcumin on the expression of cathepsins.

### Recovery of cathepsins B and L expression and proteolytic activity in curcumin-treated CCD-19Lu fibroblasts

**Figure 3:**
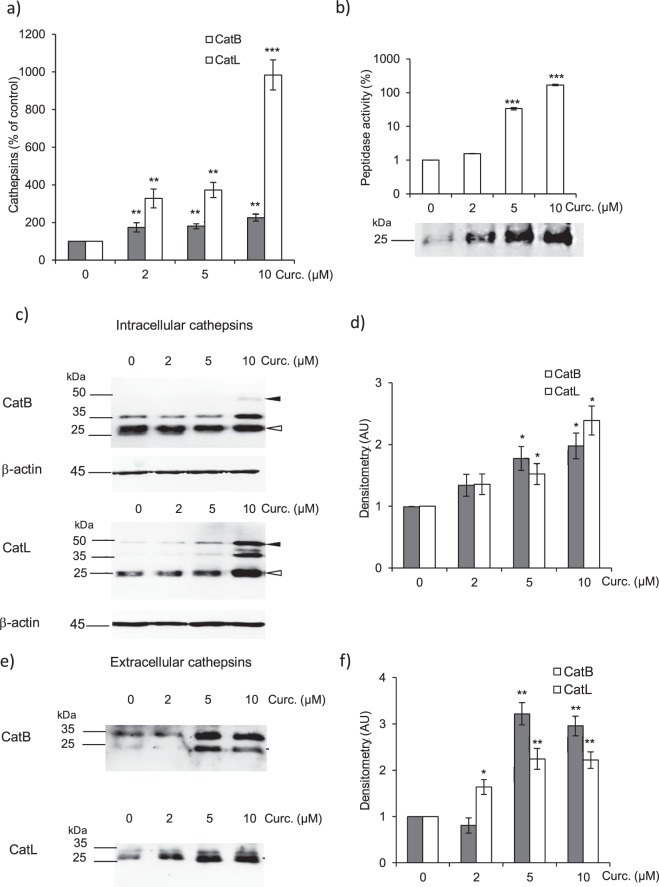
Expression level of cathepsins B and L in CCD-19Lu myofibroblasts treated by curcumin. Three days after induction of the differentiation of CCD-19l-Lu cells by TGF-β1 (5 ng/ml), curcumin (0–10 µM) was added for 48 h. (**a**) Quantitative real time PCR analysis of CatB and CatL. mRNA levels are normalized and expressed as percentage relative to untreated control (n = 3). (**b**) The related peptidase activity of secreted cysteine cathepsins was measured using Z-Phe-Arg-AMC (50 µM) as substrate. Results (corresponding to the release of fluorescent AMC, reported as arbitrary unit) are normalized and expressed as percentage relative to control in the absence of curcumin treatment (n = 3). Active site labeling of extracellular cathepsins by Biotinyl-(PEG)_2_-Ahx-LVG-DMK. Culture media of CCD-19Lu cells were incubated for 1 h with the activity-based probe (10 µM) at 30 °C according to^49^. Samples were subjected to electrophoresis on 12% SDS-PAGE under reducing conditions, electrotransferred to a nitrocellulose membrane, then blocked with 3% BSA in PBS-T. After incubation with an extravidin-peroxydase conjugate (1:3000, Sigma Aldrich) 2 h at room temperature, active cathepsins (lanes: 0, 2, 5, and 10 µM curcumin) were stained by chimiluminescence (ECL Plus Western Blotting Detection system). Full-length blots are presented in Supplementary Fig. 5. (**c**) Two days after addition of curcumin, myofibroblasts layers were lysed, and the expression of intracellular CatB and CatL was analyzed by western blotting. A representative sample is shown (n = 3). White arrows indicate mature forms; black arrows correspond to pro-CatB and pro-CatL. β-actin was used for load control. Full-length blots are presented in Supplementary Fig. 5. (**d**) Densitometric analysis of the protein level of intracellular mature CatB and CatL (normalized data relative to control without curcumin, n = 3). (**e**) Two days after treatment with curcumin, the protein level of extracellular CatB and CatL was analyzed by WB. A representative sample is shown (n = 3, white arrows, mature proteases). Full-length blots are presented in Supplementary Fig. 5. (**f**) Corresponding densitometric analysis of extracellular mature CatB and CatL. Normalized data relative to control without curcumin (n = 3).

In an initial work, we observed that mRNA levels of cathepsins B, K and L remained stable in the presence of TGF-β1, suggesting that these proteases are not transcriptionally regulated during TGF-β1-dependent myodifferentiation^29^. On the other hand, Brömme and coworkers observed that curcumin prevented collagen deposition and lung fibrosis in the murine model of bleomycin-induced fibrosis, and reported an upregulation of lung CatK^26^. Among the cysteine cathepsins showing significant ECM-degrading activities, CatK mRNA expression was increased in human and mice fibrotic lung tissues compared to normal lung specimens^47,48^. At this point, we did not observe a conclusive increase of CatK transcripts in TGF-β1-induced myofibroblasts in the presence of curcumin (Supplementary Fig. 1). This discrepancy probably stems from the fact that CCD-19Lu fibroblasts are a homogenous cell line at variance with lung biopsies that comprise a heterogeneous population of fibroblasts and differentiated myofibroblasts, but also contain bronchial and alveolar epithelial cells that constitutively express CatK. On the contrary, we observed that curcumin induced a compelling transcriptional upregulation of CatB in differentiated myofibroblasts (Fig. 3a). This increased expression was even greater for CatL (~10^3^-fold increase relative to control) following addition of curcumin (10 µM). Measurements of enzymatic activities of extracellular cysteine cathepsins in culture medium (i.e. fibroblast supernatants) revealed that this upregulation allowed the recovery of the peptidase activity of secreted cathepsins (Fig. 3b), up to levels similar to those observed in undifferentiated CCD-19Lu cells^29^. This dose-dependent rescue of cathepsin activity, as a consequence of curcumin treatment (0–10 µM), was substantiated by using the irreversible activity-based probe Biotinyl-(PEG)_2_-Ahx-LVG-DMK (with: Ahx, 6-aminohexanoic acid; PEG, polyethylene glycol, DMK, diazomethylketone) that targets the nucleophilic active site thiol of cysteine cathepsins (Fig. 3b)^49^. Western blot analysis confirmed the curcumin-induced upregulation of the protein amounts of both intracellular CatB (mainly its double-chain form) and CatL (Fig. 3c,d), along with the mature forms of secreted CatB and CatL (Fig. 3e,f). The current results are also in line with a previous report indicating that curcumin activated the protein expression of CatL in glioma cells^50^. Based on the rationale that the inhibition of extracellular ECM-degrading cathepsins relies on their regulation by secreted cystatin C during myofibrogenesis, we further investigated the pharmacological activity of curcumin with respect to the expression of cystatin C.

### Impairment of cystatin C expression in curcumin-treated CCD-19Lu myofibroblasts

**Figure 4:**
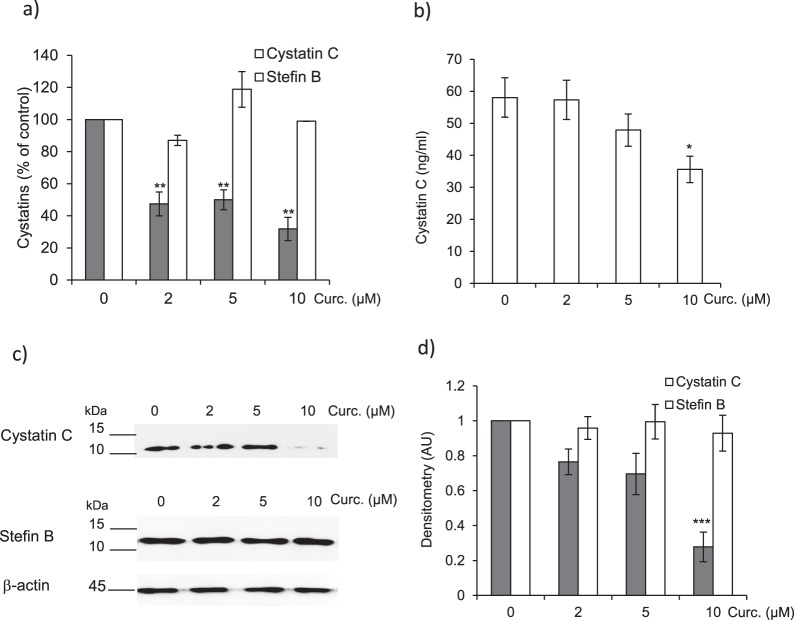
Effect of curcumin on the expression of endogenous inhibitors of cysteine cathepsins. CCD-19Lu myofibroblasts were treated with curcumin (0–10 µM) for 48 h as previously reported. (**a**) Quantitative real time PCR analysis of cystatin C and stefin B. Normalized data are expressed as percentage relative to untreated control (n = 3). (**b**) Cystatin C ELISA. After retrieval of CCD-19Lu supernatants, the concentration of secreted cystatin C was measured by sandwich ELISA (DuoSet kit, R&D Systems) (n = 3). (**c**) Culture media were concentrated (x10), submitted to a 12% SDS-PAGE electrophoresis under reducing conditions and the protein level of extracellular cystatin C was analyzed by immunoblotting. Alternatively, cell lysates were prepared and intracellular stefin B expression examined in the same way by WB. A representative sample is shown (n = 3) and β-actin was used for load control. Full-length blots are presented in Supplementary Fig. 5. (**d**) Corresponding densitometric analysis (normalized data relative to control without curcumin, n = 3).

We established earlier that, during TGF-β1-driven differentiation of human lung fibroblasts, TGF-β1 promotes secretion of cystatin C and drives the cystatin C-dependent inhibition of ECM-degrading cathepsins^29^. Conversely, we found that the expression level of intracellular stefin B (a.k.a. cystatin B) did not vary during differentiation. Here, qPCR analysis of cystatin C showed that curcumin treatment led to a >two-fold decrease of its transcription (p < 0.01) while the expression of stefin B mRNA remained unchanged (Fig. 4a). Quantification of extracellular cystatin C by a sandwich ELISA confirmed this downregulation following curcumin treatment (Fig. 4b). Immunoblot analysis of both cystatin C, the most potent circulating inhibitor of cathepsins, and stefin B, their major intracellular inhibitor, confirmed that curcumin impaired the expression of cystatin C, but had no effects on stefin B (Fig. 4c,d). In line with these results, a recent study showed that curcumin reduced cystatin C in a rat model of adenine-induced chronic kidney disease^51^. Interestingly, we observed that a significant enhancement of cystatin C occurred in bronchoalveolar fluids of IPF (idiopathic pulmonary fibrosis) patients^30^, supporting that cystatin C could be a specific and valuable biomarker of lung fibrosis. Likewise, an increased expression of cystatin C was testified for other fibrotic disorders (cardiac, liver and oral submucous fibrosis) (for review^23^). Although the exact molecular mechanisms yet remain to be clarified, this led us to propose that the rise of secreted cystatin C might favor the pathogenesis of lung fibrosis, since ECM remodeling, which depends on a subtle balance between synthesis and degradation, partly relies on cysteine cathepsins. Taken together with the drop of soluble and insoluble collagens, the present data sustained that curcumin may restore the cathepsins/cystatin C balance, both by weakening the expression of cystatin C and by rescuing the proteolytic activities of cathepsins. This therefore also raised the question of identifying likely molecular partners or cellular pathways that can make the link between the adjustment of cathepsins/cystatin C balance and the antifibrotic activity of curcumin.

### Consequences of PPARγ overexpression in curcumin-treated myofibroblasts

**Figure 5:**
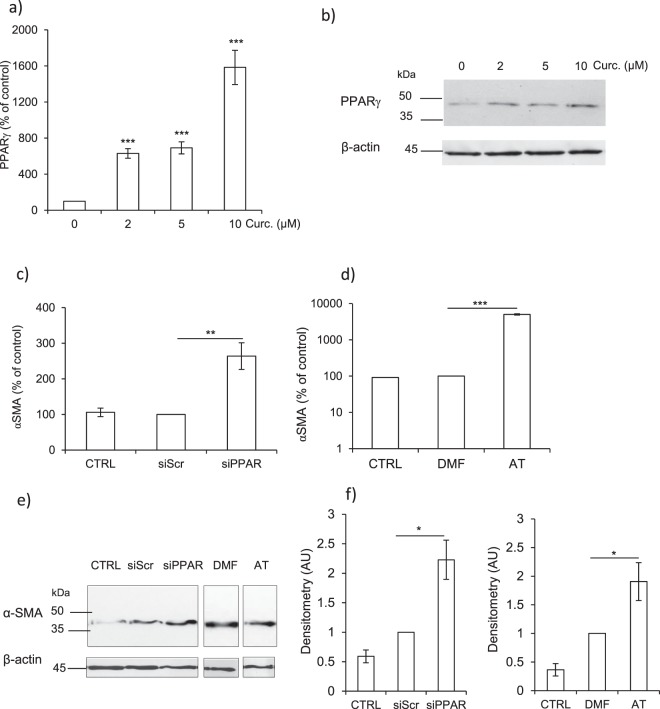
Overexpression of PPARγ in curcumin-treated myofibroblasts and consequences of PPARγ inhibition on the expression of α-SMA. CCD-19Lu myofibroblasts were treated with curcumin (0–10 µM) for 48 h as described earlier. (**a**) Quantitative real time PCR analysis of PPAR**γ**. The data are expressed as percentage relative to untreated control (n = 3). (**b**) Myofibroblasts layers were harvested and lysed, and the expression of PPAR**γ** analyzed by western blot. A representative sample of three independent experiments is shown and β-actin was used for load control. Full-length blots are presented in Supplementary Fig. 5. (**c**) Silencing of PPARγ by siRNA: six hours before addition of curcumin (10 µM), myofibroblasts were transfected with siRNA directed against PPAR**γ**. α-SMA expression was analysed by qRT-PCR (CTRL, control, i.e. curcumin, no siRNA; siScr, control (scrambled) siRNA; siPPAR, siRNA directed against PPAR**γ**; n = 3). (**d**) Pharmacological inhibition of PPAR**γ**: six hours before curcumin treatment (10 µM), the pharmacological high-affinity PPAR**γ** antagonist T0070907 (2-chloro-5-nitro-N-4-pyridinyl-benzamide, also called AT) was added to the culture medium. α-SMA expression was analyzed by qRT-PCR (CTRL, control, i.e. curcumin; DMF, vehicle; AT, cells treated with T0070907). Data are expressed as percentage relative to control (n = 3). (**e**) WB analysis of α-SMA after silencing of PPAR**γ** and chemical inhibition of PPAR**γ**. A representative sample is shown (n = 3) and β-actin was used for load control. Full-length blots are presented in Supplementary Fig. 5. (**f**) Respective densitometric analysis of WB (normalized data relative to control, n = 3).

**Figure 6:**
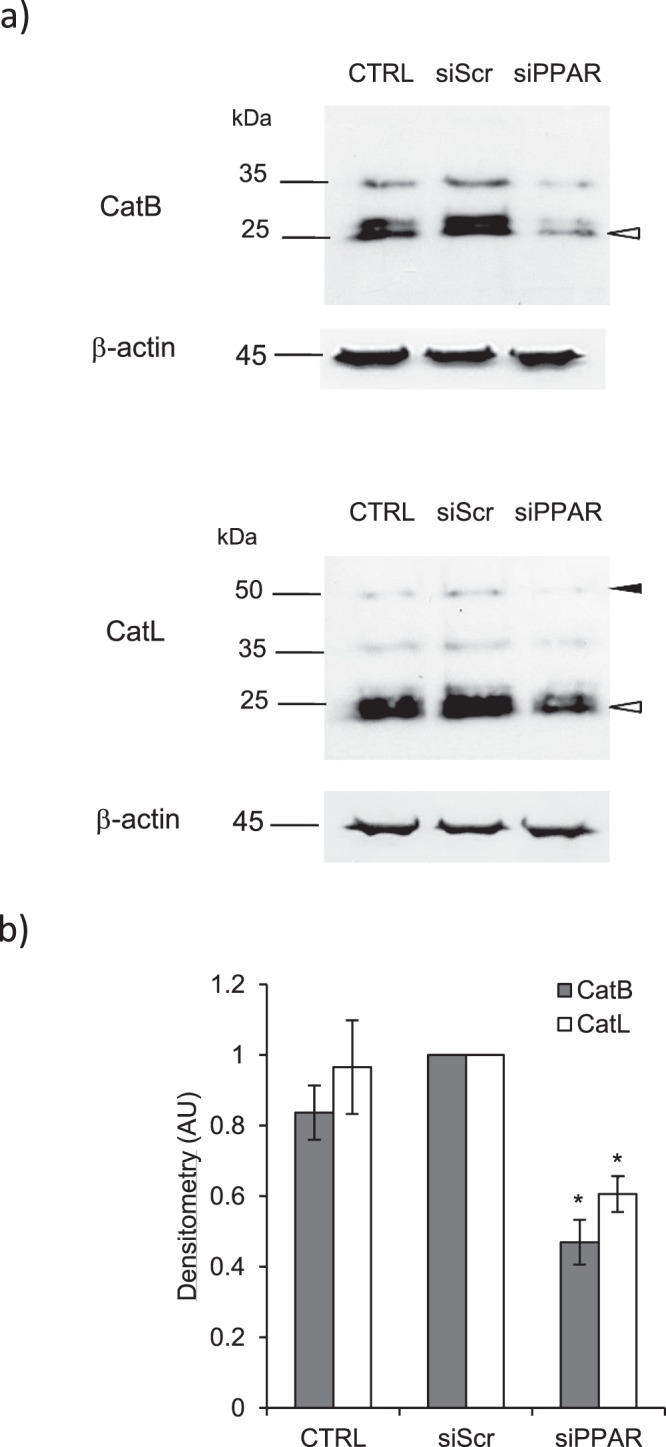
Consequences of PPARγ inhibition on the expression of cathepsins B and L. CCD-19Lu myofibroblasts were transfected with siRNA directed against PPAR**γ** (as described above) 6 h before curcumin treatment. Two days after, the expression of cathepsins was examined. (**a**) Immunoblotting analysis of intracellular CatB and CatL. White arrows indicate mature forms and black arrows indicate proforms of cathepsins. A representative sample is shown (n = 3) and β-actin was used for load control. Full-length blots are presented in Supplementary Fig. 5. (**b**) Densitometric analysis of CatB and CatL expression (Normalized Data, n = 3).

It was previously suggested that curcumin may upregulate the transcription of nuclear peroxisome proliferator-activated-receptorγ (PPARγ) (for reviews^52–54^), while a distinct study proposed that PPARγ could be involved in regulating the expression level of CatL in a monocytic cell line^55^. Also, it was observed that alcohol exposure increased the activities of both PPARγ and CatB in the rat pancreas, although no link could be pinpointed between the two molecules^56^. According to these statements, CCD-19Lu fibroblasts were treated with curcumin (0–10 µM) for 48 h as described earlier to examine PPARγ expression. Curcumin caused a robust upregulation of both PPARγ mRNA (Fig. 5a) and protein (Fig. 5b). Interestingly, previous reports are in line with our present finding that curcumin specifically upregulates PPARγ. Indeed it was demonstrated that PPARγ triggering may impair NF-κB signaling pathway in relation with a reduced phosphorylation of NF-κB at Ser536 (phospho-NF-κB, p-p65 form)^53,57,58^. Immunoblot analysis showed here that curcumin treatment led to a dose-dependent decrease in NF-κB phosphorylation while the basal level of non-phosphorylated NF-κB (p65 form) remained unchanged (Supplementary Fig. 2a,b). Then we evaluated the consequences of PPARγ inhibition on the expression level of the profibrotic marker α-SMA. Transient knockdown of PPARγ was achieved by transfection of myofibroblasts with a specific small interfering RNA (see Supplementary Fig. 3a). Alternatively, CCD-19Lu myofibroblasts were treated with 2-chloro-5-nitro-N-4-pyridinyl-benzamide (also called AT), a selective and specific PPARγ antagonist that significantly reduced (∼80%) PPARγ expression (p < 0.05) (see Supplementary Fig. 3b). Interestingly, using a rodent model of preeclampsia, treatment by 2-chloro-5-nitro-N-4-pyridinyl-benzamide led to a similar reduction of PPARγ mRNA^59^. While the expression of α-SMA remained unchanged upon treatment with a control scrambled siRNA, silencing of PPARγ induced a ~2.5 fold increase in the expression of α-SMA mRNA (Fig. 5c). Addition of 2-chloro-5-nitro-N-4-pyridinyl-benzamide (AT) resulted in a >100-fold increased transcription of α-SMA compared to PPARγ silencing (Fig. 5d). A ~two-fold increase of α-SMA protein was also observed by western blotting following either pharmacological inhibition of PPARγ or its RNA silencing (Fig. 5e,f). Likewise, the anti-fibrotic properties of curcumin were reversed by both PPARγ inhibition or its silencing by a PPARγ shRNA using renal tubular epithelial cells (HK-2 cells)^60^. Accordingly PPARγ agonists were also shown to reduce the differentiation of myofibroblasts, as well as the production of αSMA by both human lung myofibroblasts and cat corneal fibroblasts^61,62^. Likewise, transient siRNA knockdown of PPARγ induced a ~two-fold reduction of both CatB and CatL mature forms, as revealed by immunoblot analysis (Fig. 6a,b). Conversely, RNA silencing of PPARγ did not alter the expression of cystatin C mRNA (Supplementary Fig. 4a), while an ELISA-based analysis of CCD-19Lu supernatants demonstrated that the concentration of secreted cystatin C remained unchanged (Supplementary Fig. 4b). Thus, although the pathway is not yet elucidated, our results strongly suggest that the link sustaining inhibition of cystatin C by curcumin was not straightforwardly related to a molecular partnership with PPARγ. Present data disclosed that PPARγ inhibition restored TGF-β1-driven differentiation of human lung fibroblasts, and supported that curcumin-dependent triggering of PPARγ could enhance synthesis of cathepsins. The following and key question was therefore to identify the type of molecular interactions directly associating PPARγ with the overexpression of both CatB and CatL.

### Upregulation of cathepsins B and L depends on binding of PPARγ to their promoter regions

**Figure 7:**
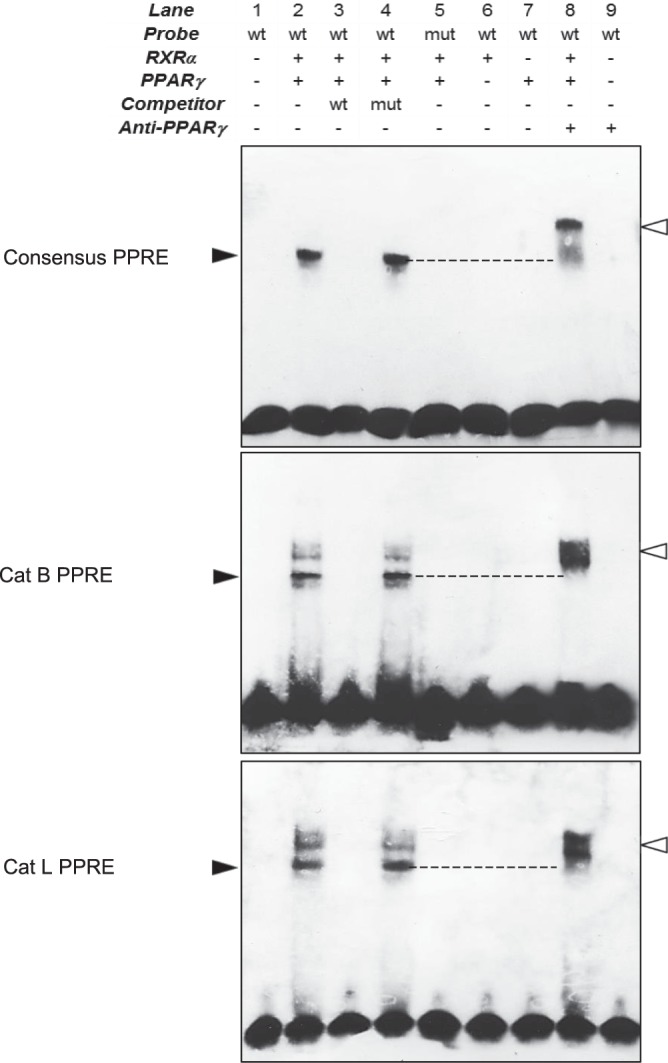
Binding of PPARγ to PPARγ response element-like (PPRE) sequences located in cathepsin B and L promoters. Electrophoretic mobility shift assays were performed using the end-labeled oligonucleotides representing the wild-type (wt) consensus PPRE, the human putative CatB PPRE-like sequences, the human putative CatL PPRE-like sequences (lanes 1, 2, 6 & 7) and mutated (mut) versions of these three sequences (lane 5) in the presence of human recombinant PPARγ and RXRα. Molar excess 200-fold (lanes 3 & 4) of unlabeled oligonucleotides was used for competition analysis. Supershift assays were performed using an anti-human PPARγ (lanes 8 & 9). Black arrows indicate specific gel shifts and white arrows indicate the supershifted bands (a representative sample is shown, n = 3). Full-length blots are presented in Supplementary Fig. 5.

Cysteine cathepsins may be controlled in various ways (namely at the transcriptional, translational, and post-translational levels), and transcriptional activation could be one of the possible mechanisms leading to cathepsin overexpression (e.g. transcription factor EB, STAT signaling pathways); also regulatory sites are found in the promoter region of some cathepsin genes including Sp1 and Sp3 binding sites, as well as interferon-stimulated response element (IRSE)^18,22,63^. The nucleotide sequences of human cathepsins were first subjected to a bioinformatic analysis (Genomatix MatInspector software, http://www.genomatix.de/; Dragon PPAR Response Element (PPRE) Spotter v.2.0, http://www.cbrc.kaust.edu.sa/ppre/) that allowed us to identify putative PPRE-like sequences in the promoter regions of both CatB and CatL (confidence score >0.9). Conversely, we did not identify hypothetical PPRE sequences in the CatK promoter region, in agreement with the lack of upregulation for CatK in fibroblasts. Consistently the expression of CatK is primarily modulated by RANKL (receptor activator of NF-κB ligand) (see^64^). The functionality of the PPRE-like sequences for CatB and CatL was further assessed by an electrophoretic mobility shift assay (EMSA), using human recombinant PPARγ and RXRα (Retinoid X Receptor-α). Incubation of biotin-labeled CatB- or CatL-PPRE oligonucleotides with recombinant PPARγ and RXRα resulted in the formation of complexes with reduced electrophoretic mobility (Fig. 7, lane 2). These specific complexes were not observed with the mutated PPRE-like oligonucleotides (Fig. 7, lane 5) that were used as negative controls. The binding specificity of PPARγ to these PPRE sites was validated by a competitive inhibition assay with unlabeled wild-type oligonucleotides (Fig. 7, lane 3); conversely unlabeled mutated CatB-PPRE and CatL-PPRE oligonucleotides did not prevent PPARγ binding (Fig. 7, lane 4). Additionally supershift assays were performed using an anti-human PPARγ antibody, and assessed by the binding of PPARγ and RXRα to labeled consensus PPRE (positive control^65,66^). The presence of a supershifted complexes demonstrated that both PPARγ and RXRα bound as a heterodimer to the PPRE-like sequences of the promoter regions (Fig. 7, lane 8). Of note, we consistently observed two shifted complexes with the CatB- and CatL-derived PPRE-like sequences. It could suggest the presence of distinct modes of binding with different stoechiometries^67^, which might be allowed by the greater length of the PPRE-like sequences relative to the consensus PPRE sequence that we used as positive control. Taken together, these data established that PPRE-like sequences in the promoter regions of both CatB and CatL were functional for their binding to PPARγ and RXRα, suggesting that this binding could elicit their transcription. Finally, present results also supported that anti-fibrotic effects of curcumin were partly mediated by the PPARγ-driven upregulation of matrix-degrading proteases, CatB and CatL.

### Concluding remarks

**Figure 8:**
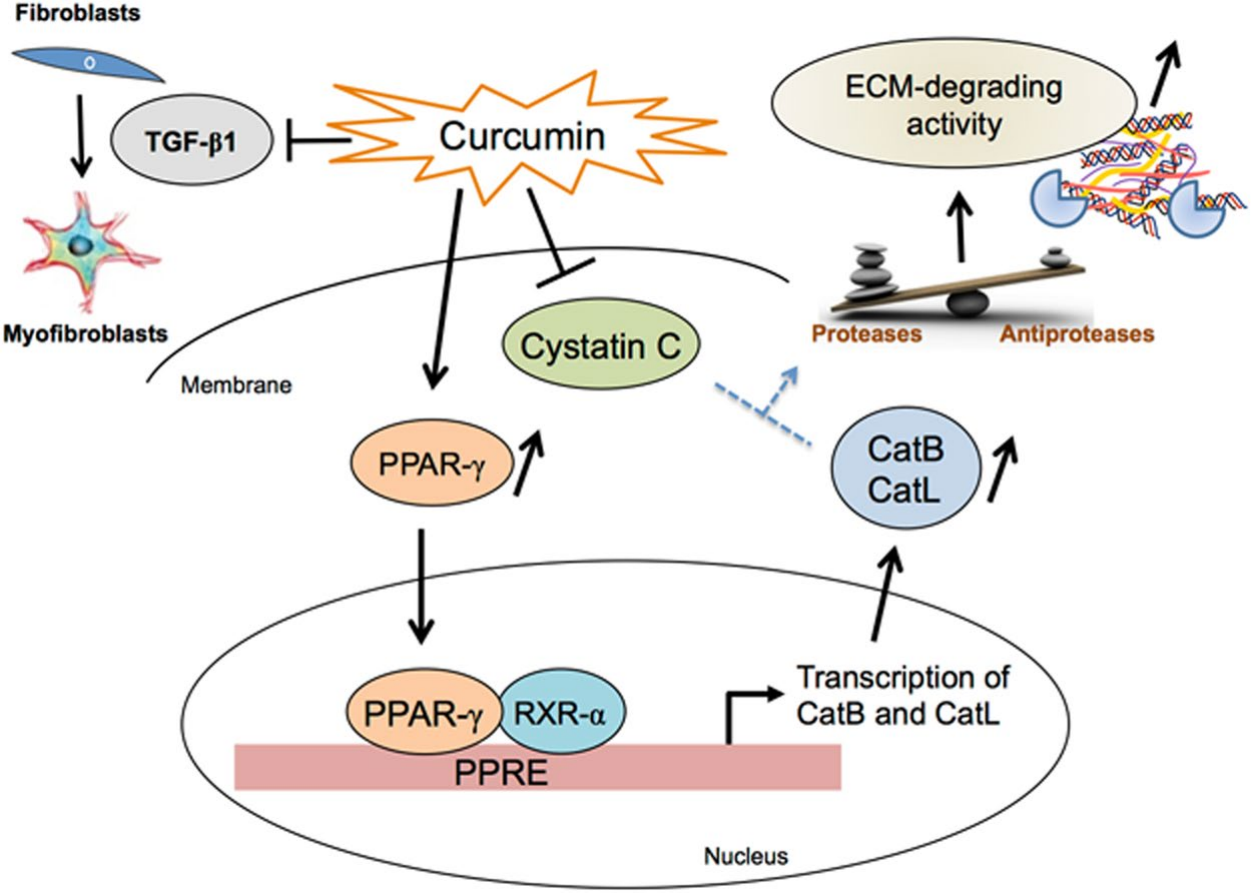
Anti-fibrotic properties of curcumin are associated with PPARγ-driven upregulation of cathepsins B and L (a synthetic drawing). Curcumin inhibits the differentiation of lung CCD-19Lu fibroblasts (as confirmed by decreased amounts of both insoluble and soluble type I collagen, as well the impairment of expression levels of α-SMA, a profibrotic biomarker and endogenous TGF-β1). Moreover, curcumin triggers PPARγ that in turn may bind to PPARγ response element-like sequences, which are located in the promoter regions of CatB and CatL, and drives the transcription and expression of the two proteases. Otherwise curcumin down-regulates the expression level of cystatin C, the most potent circulating inhibitor of secreted cathepsins. Taken together this could allow the recovery of the proteolytic activity of secreted cathepsins and the restoration of the “cathepsins/cystatin C” balance, thus promoting ECM-degrading properties of cathepsins.

During these last few years we have paid a peculiar attention to the understanding of the role of cysteine cathepsins (primarily cathepsins B, L and K) in fibrotic processes. We established that TGF-β1 induces the secretion of cystatin C that - in turn - impairs the activity of extracellular matrix-degrading cathepsins, by using primary fibroblasts from IPF patients and a validated model of human lung CCD-19Lu fibroblasts. We also demonstrated a relevant increase of cystatin C in IPF bronchoalveolar lavage fluids that may reflect dysregulation of proteolytic activity in lung, and proposed that cystatin C could be used as a clinical biomarker of lung fibrosis. Otherwise Brömme and his collaborators demonstrated that curcumin, a potent anti-inflammatory and anti-proliferative nutraceutical, is an effective anti-fibrotic compound, using the murine model of bleomycin-induced lung fibrosis^26^. However, signaling pathways and accurate molecular mechanisms are still poorly described. Here we confirmed that curcumin inhibited TGF-β1-dependent lung fibroblast differentiation of human lung CCD-19Lu fibroblasts. Accordingly, the expression of the profibrotic marker α-SMA was down regulated, and the levels of both soluble and insoluble collagen were decreased to values similar to those observed with undifferentiated fibroblasts. Otherwise curcumin impaired the amount of secreted cystatin C, but not stefin B, and up-regulated the mRNA and protein expression levels of both CatB and CatL, leading to a restoration of the “cathepsins/cystatin C balance”. Consequently, curcumin could promote the ECM-degrading activities of cathepsins, thereby relieving the detrimental accumulation of extracellular matrix. Potential molecular pathways were further examined. We found that curcumin participates to the up-regulation of nuclear PPARγ and identified functional PPARγ response element-like sequences in the promoter regions of both cathepsins B and L, that may elicit their transcriptional activation (see Fig. 8: synthetic diagram). However, curcumin is a multifaceted molecule that may interact with various molecular partners. For instance, the anti-inflammatory activity of curcumin is associated to the repression of signalling pathways including NF-κB, STAT3, Nrf2, and COX-2 (for review^68^). In the present work, we have partially deciphered its pharmacological mechanism of action on human lung fibroblasts. Nevertheless, taking into account its versatile properties, we cannot exclude that its anti-fibrotic properties, which as demonstrated here closely relate to PPARγ activation that in turn triggers the expression of cathepsins, also involve other cellular signaling pathways.

## Methods

### Antibodies

The antibodies used for immunoblot analysis, immunofluorescent labeling or electrophoretic mobility shift assay (EMSA) were as follows: mouse anti-α-smooth muscle actin (α-SMA) (1:1000 for western blot and 1:100 for immunofluorescence) and mouse anti-β-actin (1:1000) were supplied by Sigma-Aldrich (Saint Quentin Fallavier, France). Mouse anti-human TGF-β1 (1:1000) was purchased from Abcam (Cambridge, UK). Goat anti-human CatB and CatL (1:1000), mouse anti-human cystatin C (1:400) and goat anti-human cystatin (stefin) B (1:1000) were from R&D systems (Minneapolis, MN, USA). Rabbit anti-human NF-κB-p65 (1:500) and phospho-NF-κB-p65 (p-p65) (1:500) were from Cell Signaling Technology (Beverly, MA, USA). Mouse anti-PPARγ used for Supershift EMSA was from Santa Cruz Biotechnology Inc. (Heidelberg, Germany) and rabbit anti-PPARγ used for western blot (1:500) was from Novus Biologicals, Inc. (Littleton, CO, USA). Goat anti-rabbit IgG-peroxidase conjugate, goat anti-mouse IgG-peroxidase conjugate, and rabbit anti-goat IgG-peroxidase conjugate were from Sigma-Aldrich.

### Chemicals, pharmacological inhibitors and reagents

Curcumin, 2-chloro-5-nitro-N-4-pyridinyl-benzamide (PPAR**γ** antagonist T0070907, AT), L-3-carboxy-trans-2, 3-epoxy-propionyl-leucylamide-(4-guanido)-butane (E-64), pepstatin A, ethylene diamine tetra acetic acid (EDTA), 4-(2-aminoethyl) benzenesulfonyl fluoride hydrochloride (Pefabloc), S-methyl thiomethanesulfonate, polyethylene glycol lauryl ether (Brij35), dithiothreitol (DTT) and N,N-dimethylformamide (DMF) were obtained from Sigma-Aldrich. Biotinyl-(PEG)_2_-Ahx-LVG-DMK was synthetized as previously described (mass spectrometry: theoretical Mw, m/z = 854.07; experimental Mass, m/z = 853.47)^49^. Benzyloxy-carbonyl-Phe-Arg-7-amino-4-methyl coumarin (Z-FR-AMC) was purchased from R&D systems.

### Treatments of CCD-19Lu fibroblasts by curcumin

The CCD-19Lu normal human lung cell line was purchased from the American Type Culture Collection (ATCC, Manassas, VA, USA). The fibroblasts were cultured in completed Eagle’s minimum essential medium supplemented with 10% heat-inactivated fetal calf and 1% penicillin/streptomycin (LGC Standards SARL, Molsheim, France) at 37 °C, in an atmosphere containing 5% CO_2_. Cells were cultured up to passage 5. Cells were plated at 200,000 cells into 6-well plates, cultured for 24 h and were starved overnight in serum-free medium. Differentiation of myofibroblasts was induced by addition of recombinant TGF-β1 (5 ng/ml, R&D systems) and allowed to incubate for 3 days. Controls were similarly incubated in the absence of TGF-β1. Then curcumin (0–50 µM) was added at day 3 to myofibroblasts and incubated for 48 h.

### Cell viability assay

CellTiter 96® AQ_ueous_ One Solution Cell Proliferation Assay (MTS) kit (Promega, Charbonnière-Les-Bains, France) was used to evaluate cell viability according to the manufacturer’s instructions. Briefly, cells were plated in 96-well plates containing 100 µl of medium and treated with TGF-β1, then curcumin (0–50 µM) as described above. At various time (24–96 h), the MTS reagent (20 µl/well) was added and cells were incubated at 37 °C for 3 h. The absorbance was measured at 490 nm (Microplate Reader VersaMAx, Molecular Devices, St Grégoire, France). All the experiments were repeated three times. MTS assay results were normalized using as reference the proliferation rate of CCD-19Lu cells cultured in the absence of curcumin (control value defined as 100%).

### Immunofluorescence

Control and treated CCD-19Lu cells were seeded into 8-well LabTek chamber slides. Cells were fixed in 4% paraformaldehyde and permeabilized with 0.3% TritonX-100 in PBS as described previously^29^. Photomicrographs were acquired by using an inverted fluorescence microscope (EVOS fl) from Advanced Microscopy Group (Mill Creek, WA, USA) at 200× magnification.

### Western blot analysis

At each time point, culture media were harvested in the preservative buffer A (0.1 M sodium acetate buffer, pH 5.5), containing protease inhibitors (0.5 mM Pefabloc, 0.5 mM EDTA, 1 mM *S*-methyl thiomethanesulfonate, 0.04 mM pepstatin A). The culture medium was centrifuged for 5 min (10,000 g, 4 °C) to remove cell debris and concentrated 50-fold (Vivaspin concentrator tube, exclusion limit 2,000, Sartorius AG, Göttingen, Germany). Cell layers were washed once in ice-cold PBS and harvested by scraping in ice-cold buffer A. Following three freeze-thaw cycles using liquid nitrogen, soluble proteins were retrieved by centrifugation for 10 min (10,000 *g*, 4 °C). The remaining membrane pellets were resuspended in buffer A and stored at −80 °C. Protein concentrations were determined by Bradford assay (Bio-Rad). Samples were prepared in Laemmli buffer and boiled for 5 min. Concentrated culture media (60 μg/well) and cell layer lysates (10 μg/well) were loaded onto 12% SDS-PAGE, and electrophoresis was carried out under reducing conditions. Prestained molecular weight standards (Precision Plus Protein Standards) were supplied by Bio-Rad. The separated proteins were transferred to a nitrocellulose membrane (Amersham Biosciences, Buckinghamshire, UK). The membranes were blocked with 5% nonfat powdered milk in PBS, 0.1% Tween 20 (PBS-T). Following incubation with the primary antibodies (overnight at 4 °C under agitation), the secondary antibodies (1:5000) were added for 1 h at room temperature. Proteins were visualized by chemiluminescence (ECL Plus Western blotting detection system; Amersham Biosciences) according to the manufacturer’s instructions. Constant loading in proteins was checked by incubation with a monoclonal anti-β-actin antibody. Bands were quantified by densitometric analysis using the ImageJ software (National Institutes of Health, Bethesda, MD, USA).

### Analysis of RNA

Total RNA was isolated from cell lysate using GeneJET RNA Purification Kit (Thermo Fisher Scientific, Fermentas, Illkirch, France). cDNA was synthetized using 0.5 µg of RNA and RevertAid H minus M-MuLV reverse transcriptase (Fermentas). The quantitative analysis of transcripts of αSMA, TGF-β1, COL1A1, COL1A2, CATB, CATL, CSTC, CSTB and PPAR**γ** was performed with the MyiQ system (Bio-Rad) using the Absolate SYBR Green fluorescent mix (Dominique Dutscher, Brumath, France). For quantification of relative expression levels, the ΔΔCt method was used (normalization gene, human ribosomal protein S16 (RPS16)).

Human primers sequences used are as follows: αSMA (5′-CAGGGCTGTTTTCCCATCCAT-3′; 5′-GCCATGTTCTATCGGGTACTTC-3′), TGF-β1 (5′-CTAATGGTGGAAACCCACAACG-3′; 5′-TATCGCCAGGAATTGTTGCTG-3′); COL1A1 (5′-GTGCGATGACGTGATCTGTGA-3′; 5′-CGGTGGTTTCTTGGTCGGT-3′), COL1A2 (5′-TCCAAGGACAAGAAACAC-3′; 5′-GCAGCCATCTACAAGAAC-3′), CATB (5′-AGAGTTATGTTTACCGAGGACCT-3′; 5′-GCAGATCCGGTCAGAGATGG-3′), CATL (5′-GGAAAACTGGGAGGCTTATCTC-3′; 5′-AGCATAATCCATTAGGCCACCA-3′), Cystatin C (5′-GATCGTAGCTGGGGTGAACT-3′; 5′-CCTTTTCAGATGTGGCTGGT-3′), Stefin B (5′-TGTCATTCAAGAGCCAGGTG-3′; 5′-AGCTCATCATGCTTGGCTT-3′); PPARγ (5′-GGGATCAGCTCCGTGGATCT-3′; 5′-TGCACTTTGGTACTCTTGAAGTT-3′), RPS16 (5′-ACGTGGCCCAGATTTATGCTAT-3′; 5′-TGGAAGCCTCATCCACATATTTC-3′).

### Measurement of cysteine cathepsins activity

Peptidase activities of cathepsins were measured at 37 °C in concentrated culture media. Measurements were performed in 0.1 M sodium acetate buffer, pH 5.5, 5 mM DTT, 2 mM EDTA, and 0.01% Brij35 using Z-Phe-Arg-AMC as substrate (50 µM) (λ exc, 350 nm; λ em, 460 nm). Assays were performed in 96-well plates (Nunc A/S, Roskilde, Denmark) with a Gemini spectrofluorimeter (Molecular Devices). Control experiments were performed in the presence of E-64.

### Labeling of active cathepsins by a biotinylated peptidyl diazomethylketone

Culture media of CCD-19Lu cells were incubated in the assay buffer (see the former paragraph) for 1 h at 30 °C with Biotinyl-(PEG)_2_-Ahx-LVG-DMK (10 µM), an irreversible activity-based probe, where the peptidyl moiety (i.e. Leu-Val-Gly) corresponds to the N-terminal substrate-like segment of human cystatin C^49^. Samples were subjected to electrophoresis on 12% SDS-PAGE under reducing conditions. Then, proteins were electrotransferred to a nitrocellulose membrane, blocked with 3% BSA in PBS-T and incubated with an extravidin-peroxydase conjugate (1:3000, Sigma Aldrich) 2 h at room temperature. Proteins were vizualized by chimiluminescence (ECL Plus Western Blotting Detection system).

### Inhibition of PPARγ expression by siRNA or pharmacological inhibition

PPAR**γ** small interfering RNA (siPPAR**γ**, SC-29455) or scrambled control siRNA (siScr, SC-37007) were obtained from Santa Cruz. CCD-19Lu cells were seeded into 6-well plates as described above. Six hours before curcumin treatment, the myofibroblasts were transfected with 6 nM siRNA in basal medium using HiPerFect transfection Reagent (Qiagen SAS, Courtaboeuf, France). Alternatively, CCD-19Lu cells were treated by the PPAR**γ** antagonist (T0070907, 1 µM) or control solution (DMF). Forty-eight hours after curcumin treatment, both cell lysates and culture media were retrieved for RNA and protein isolation.

### Collagen and cystatin C assays

Total soluble collagen in cell culture supernatants (soluble collagen) and cell layer lysates (insoluble collagen) was quantified using the Sircol collagen assay following the manufacturer’s instructions (Biocolor, Belfast, UK). Briefly, Sirius red reagent (1 ml) was added to concentrated culture media or cell lysates (100 μl) and mixed for 30 minutes. The collagen–dye complex was precipitated by centrifugation at 10,000 *g* for 10 minutes, washed with ethanol, and dissolved in 0.5 M NaOH. Finally, the absorbance was determined at 540 nm (microplate reader VersaMax, Molecular Devices). The concentration of human cystatin C in culture supernatants was determined by sandwich ELISA (DuoSet kit, R&D Systems) according to^30^.

### Electrophoretic mobility shift assay (EMSA)

The putative Cat B and Cat L PPRE-like elements were identified using Genomatix MatInspector software (Genomatix Software, Ann Arbor, MI, USA) (http://www.genomatix.de/) and Dragon PPAR Response Element (PPRE) Spotter v.2.0 (http://www.cbrc.kaust.edu.sa/ppre/). Their mutant sequences (mt) were designed and verified by these softwares. A consensus PPARγ response element was used as control^66^. The oligonucleotides used were as follows: CAT B-PPRE 5′-TCTCCTGACCTGTGATCCGCCCGCCTCG-3′; CAT B-PPREmt 5′-TCTCCTGATGTAGGATACTCCTGCCTCG-3′; CAT L-PPRE 5′-TTATGGGGTAAAGGCAGAGGTAATTATT-3′; CAT L-PPREmt 5′-TTATGCGGTACCCGCTTCGGTAATTATT-3′; consensus PPRE 5′-CAAAACTAGGTCAAAGGTCA-3′ and consensus PPREmt 5′-CAAAACTAGCACAAAGCACA-3′. Mutated bases are underlined. The oligonucleotide probes were synthetized (Thermo Fisher Scientific) and labeled using 3′End DNA labeling Kit following the manufacturer’s recommendations (Thermo Fisher Scientific). EMSA was performed according to manufacturer’s instructions. After annealing the complementary oligonucleotides, binding reactions were performed using the LightShift Chemiluminescent EMSA Kit (Thermo Fisher Scientific). Labeled DNA (4 fmol) was incubated on ice for 20 min with recombinant human PPARγ (3.7 pmol) and RXRα (3.9 pmol) (OriGene Technologies, Inc., Rockville, MD, USA) in a final volume of 20 μl of binding buffer containing 20 ng of Poly (dI.dC), 2.5% glycerol, 0.05% NP-40, MgCl2 5 mM. For competition assays, 800 fmol of unlabeled wild type or mutant oligonucleotides was added to the reaction mixture. For supershift assays, 1 μg of human PPARγ antibody was preincubated with the proteins for 15 min before oligonucleotide addition. All samples were fractionated for 45 min at 100 V in a 4% non-denaturing polyacrylamide gel containing 1x Tris borate-EDTA at 4 °C. DNA was then transferred to Biodyne Precut Nylon Membrane (Thermo Fisher Scientific), UV cross-linked, probed with streptavidin-horseradish peroxidase conjugate and incubated with LightShift chemiluminescent substrate (Pierce).

### Statistical analysis

All statistical analyses of data were performed using a non-parametric test (Kruskal-Wallis). The values represent means +/− sem (three independent experiments, n = 3). Examination of the means and medians showed that they were close together, implying that their values were symmetrically distributed around the central tendency. Accordingly, the means +/− sem values were used. (*p < 0.05; **p < 0.01; ***p < 0.001).

## Supplementary information

Supplementary files
