# Supplementary material for: Curcumin inhibits the TGF-β1-dependent differentiation of lung fibroblasts via PPARγ-driven upregulation of cathepsins B and L
Source: Sci Rep. 2019 Jan 24;9:491. doi: 10.1038/s41598-018-36858-3 (PMC6345753; doi:10.1038/s41598-018-36858-3)
Supplement: Supplementary file 1 — Supplementary files [file 41598_2018_36858_MOESM1_ESM.pdf]

Expression level of cathepsin K in CCD-19Lu myofibroblasts treated by curcumin.

Manuscript: Curcumin inhibits the TGF-β1-dependent differentiation of lung fibroblasts via PPARγ-driven upregulation of cathepsins B and L.

Authors: Ahlame Saidi, Mariana Kasabova, Lise Vanderlynden, Mylène Wartenberg, Ghania Hounana Kara-Ali, Daniel Marc, Fabien Lecaille and Gilles Lalmanach.

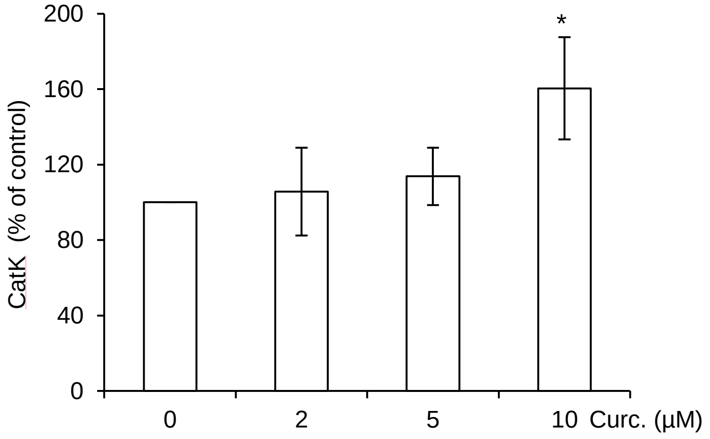

Supplementary figure 1: Expression level of cathepsin K in CCD-19Lu myofibroblasts treated by curcumin: Three days after induction of the differentiation of CCD-19-Lu cells by TGF-β1 (5ng/ml), curcumin (0-10 µM) was added for 48h. CatK transcripts were analyzed by quantitative real time PCR. mRNA levels are normalized and expressed as percentage relative to control without curcumin (n=3).

Consequences of curcumin treatment on NF-κB activation.

Manuscript: Curcumin inhibits the TGF-β1-dependent differentiation of lung fibroblasts via PPARγ-driven upregulation of cathepsins B and L.

Authors: Ahlame Saidi, Mariana Kasabova, Lise Vanderlynden, Mylène Wartenberg, Ghania Hounana Kara-Ali, Daniel Marc, Fabien Lecaille and Gilles Lalmanach.

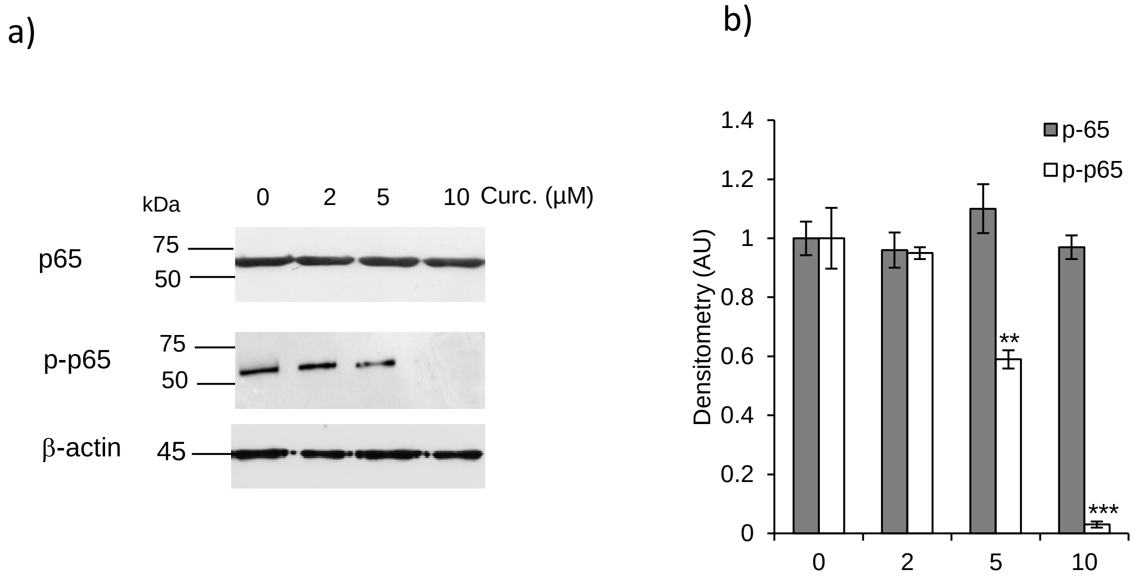

Supplementary figure 2: Consequences of curcumin treatment on NF-κB activation. Three days after induction of the differentiation of CCD-19Lu cells by TGF-β1 (5ng/ml), curcumin (0-10 µM) was added for 48 h. Myofibroblasts layers were treated as described in details in the experimental section, except that (i) they were lysed in a preservation buffer (PhosphoSafe, Merck, Darmstadt, Germany) in the presence of the phosphatase inhibitor cocktail 3 (Sigma), and (ii) an additional sonication step (20 s) was performed to disrupt DNA. a) The expression of unphosphorylated NF-κB-p65 (p65) and its phosphorylated form (phospho-NF-κB-p65; so called p-p65) was analyzed by western blot. β-actin was used for load control. Full-length blots are presented in supplementary figure 5. b) Corresponding densitometric analysis of p65 and p-p65 (p-65, grey bar; p-p65, white bar). Normalized data relative to control without curcumin (n=3).

Characterization of genetic and chemical PPARγ inhibition.

Manuscript: Curcumin inhibits the TGF-β1-dependent differentiation of lung fibroblasts via PPARγ-driven upregulation of cathepsins B and L.

Authors: Ahlame Saidi, Mariana Kasabova, Lise Vanderlynden, Mylène Wartenberg, Ghania Hounana Kara-Ali, Daniel Marc, Fabien Lecaille and Gilles Lalmanach.

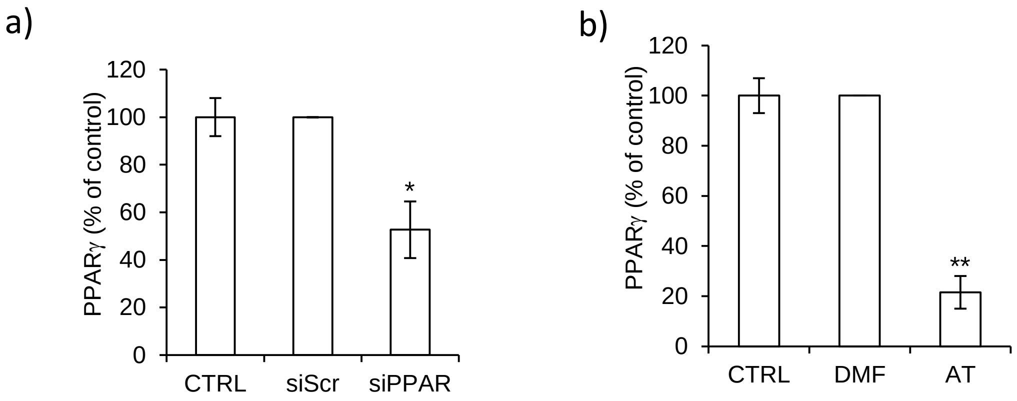

Supplementary figure 3: Characterization of genetic and chemical PPARγ inhibition: a) Silencing of PPARγ: six hours before addition of curcumin (10 µM), CCD-19Lu differentiated myofibroblasts were transfected with siRNA (CTRL, control curcumin without siRNA; siScr, control siRNA; siPPAR, siRNA directed against PPARγ). Two days after treatment, a quantitative real time PCR analysis of PPARγ was completed. Data are expressed as percentage relative to siScr control (n=3). b) Pharmacological inhibition of PPARγ: alternatively, six hours before curcumin treatment (10 µM), the cell-permeable, specific and high-affinity PPARγ antagonist 2-chloro-5-nitro-N-4-pyridinyl-benzamide (T0070907) and so called AT was added to the culture medium (CTRL, control, i.e. curcumin; DMF, cells incubated with DMF (vehicle); AT, cells treated with antagonist T0070907). 48 h after treatment with AT, the expression level of PPARγ was determined by quantitative real time PCR analysis. The data are expressed as percentage relative to control (n=3).

Consequences of PPARγ inhibition on the expression of cystatin C.

Manuscript: Curcumin inhibits the TGF-β1-dependent differentiation of lung fibroblasts via PPARγ-driven upregulation of cathepsins B and L.

Authors: Ahlame Saidi, Mariana Kasabova, Lise Vanderlynden, Mylène Wartenberg, Ghania Hounana Kara-Ali, Daniel Marc, Fabien Lecaille and Gilles Lalmanach.

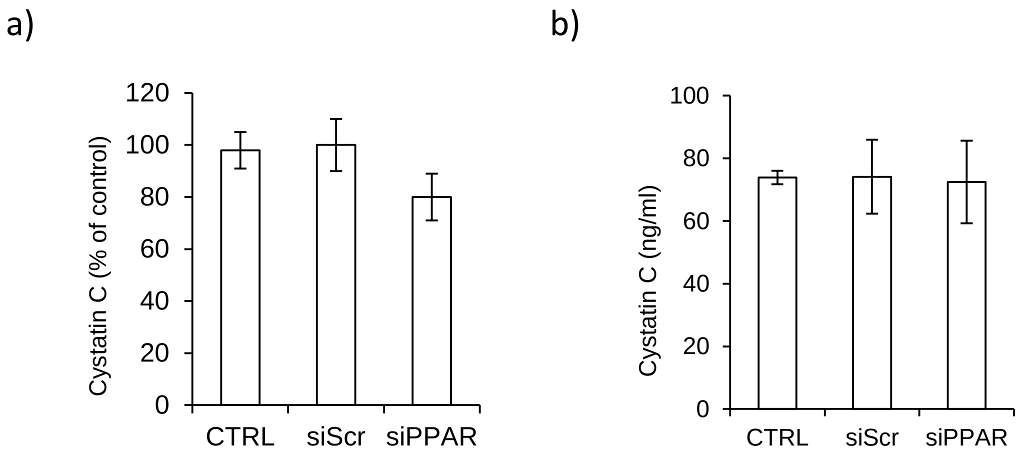

Supplementary figure 4: Consequences of PPARγ inhibition on the expression of cystatin C. Six hours before addition of curcumin (10 µM) to CCD-19Lu myofibroblasts, cells were transfected with siRNA (CTRL, control, i.e. curcumin; siScr, control (scrambled) siRNA; siPPAR, siRNA directed against PPARγ**)** and incubated for 2 days. a) Cystatin C expression was analyzed by qRT-PCR. Data are expressed as percentage relative to control (n=3). b) Cystatin C immunoassay: CCD-19Lu supernatants (culture media) were collected and the concentration of secreted cystatin C was measured by ELISA (DuoSet, R&D Systems). CTRL, control, i.e. curcumin; siScr, control (scrambled) siRNA; siPPAR, siRNA directed against PPARγ (n=3).

Full-length gels and blots.

Manuscript: Curcumin inhibits the TGF-β1-dependent differentiation of lung fibroblasts via PPARγ-driven upregulation of cathepsins B and L.

Authors: Ahlame Saidi, Mariana Kasabova, Lise Vanderlynden, Mylène Wartenberg, Ghania Hounana Kara-Ali, Daniel Marc, Fabien Lecaille and Gilles Lalmanach.

Supplementary file 5:

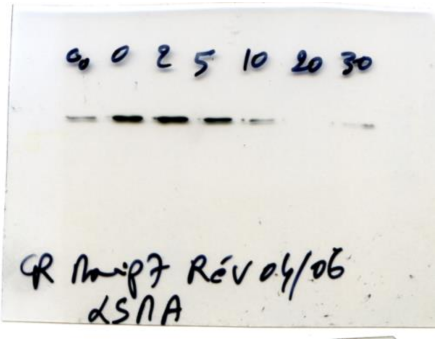

Figure 1c, αSMA

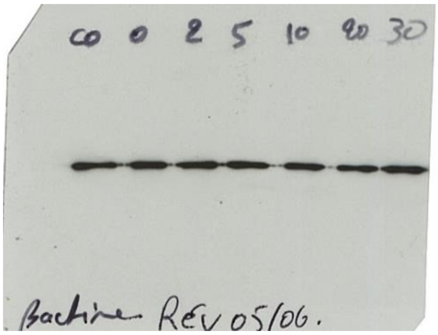

Figure 1c, β-actin

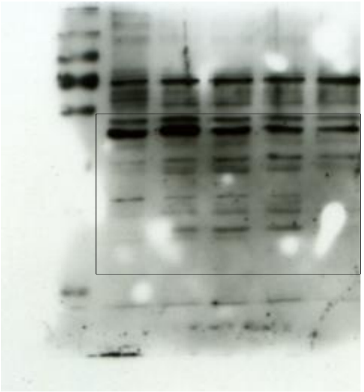

Figure 2b, TGF-β1

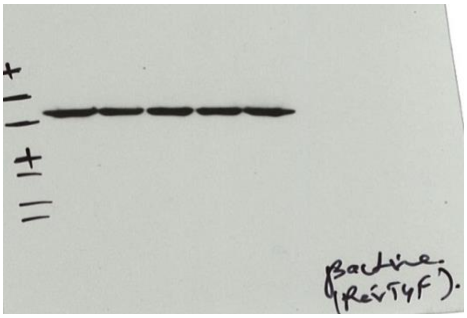

Figure 2b, β-actin

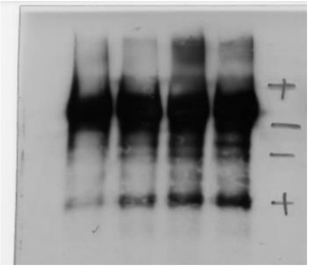

Figure 3b, activity-based probe

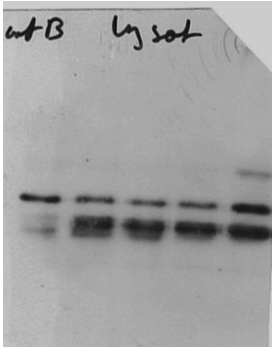

Figure 3c, CatB

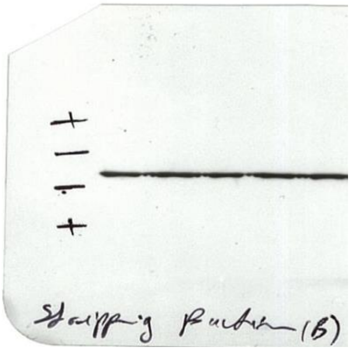

Figure 3c, β-actin

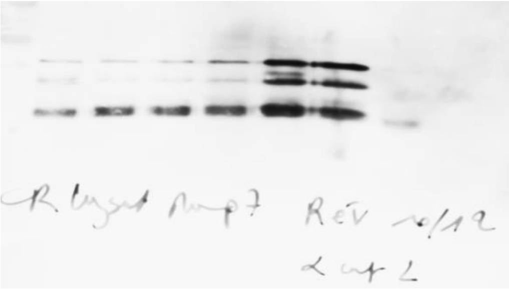

Figure 3c, CatL

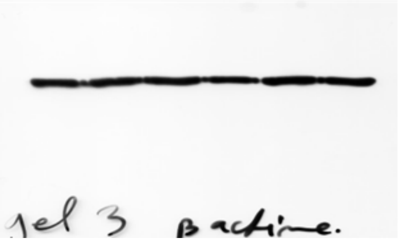

Figure 3c, β-actin

Pro-CatB

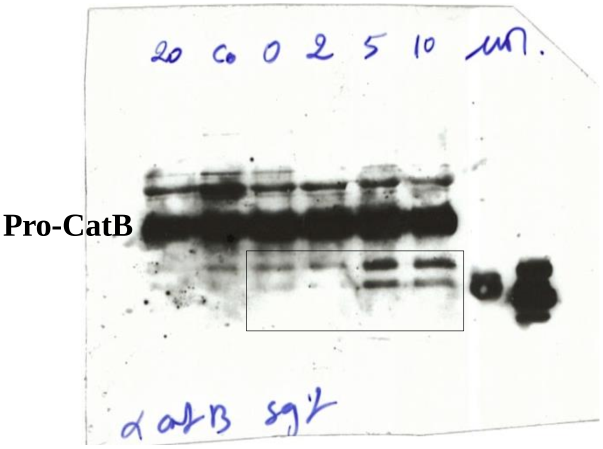

Figure 3e, CatB (mature form)

Pro-CatL

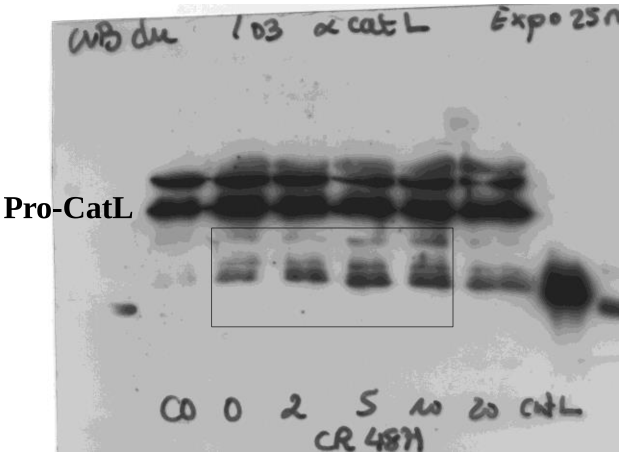

Figure 3e, CatL (mature form)

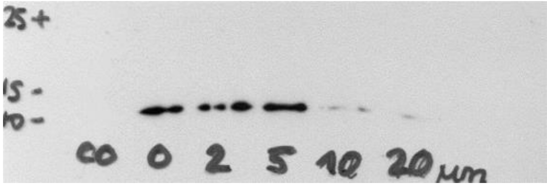

Figure 4c, cystatin C

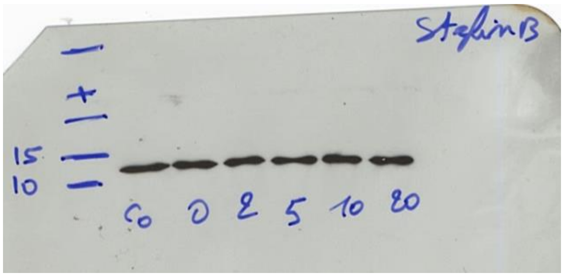

Figure 4c, stefin B

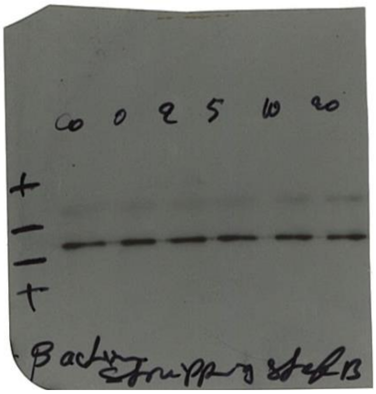

Figure 4c, β-actin

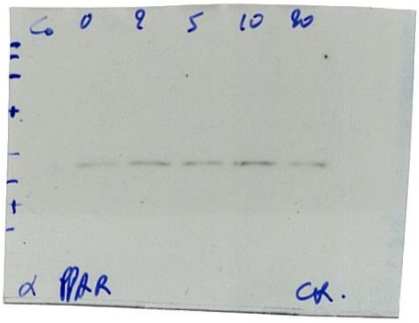

Figure 5b, PPARγ

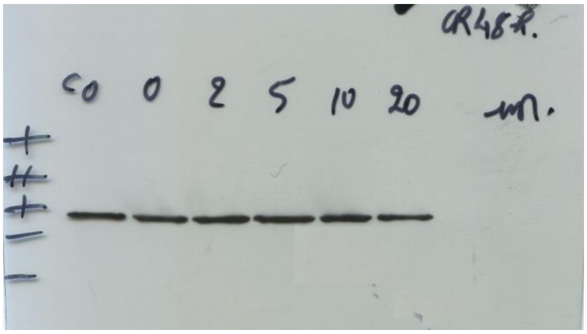

Figure 5b, β-actin

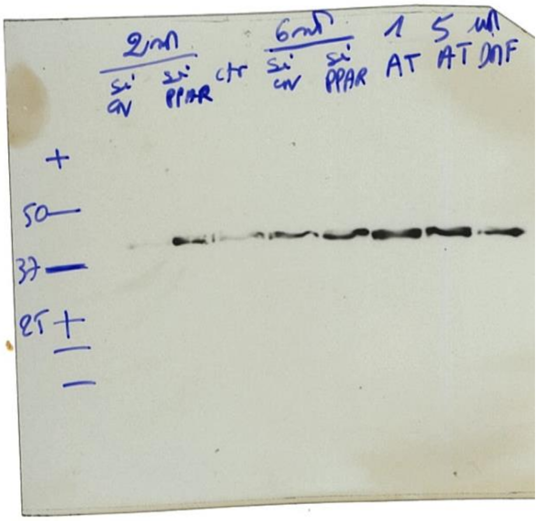

Figure 5e, αSMA

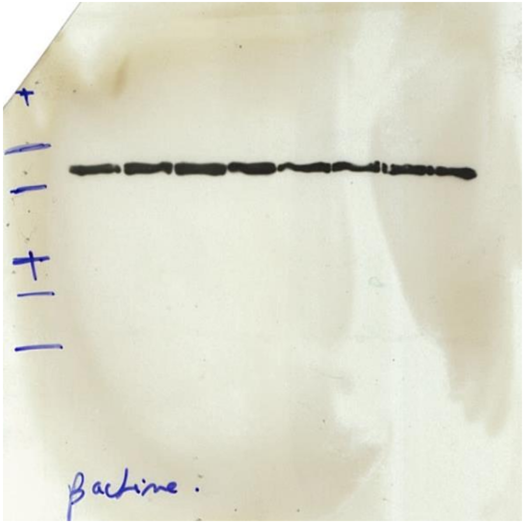

Figure 5e, β-actin

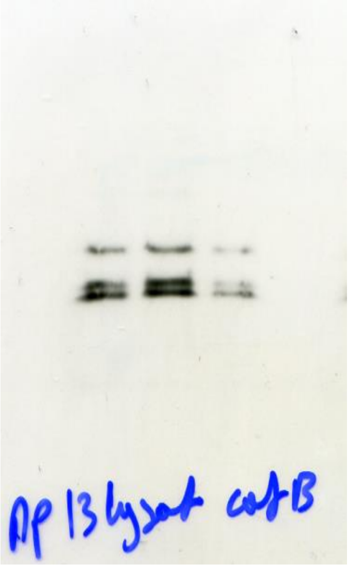

Figure 6a, CatB

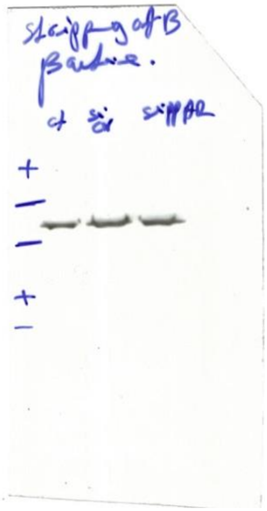

Figure 6a, β-actin

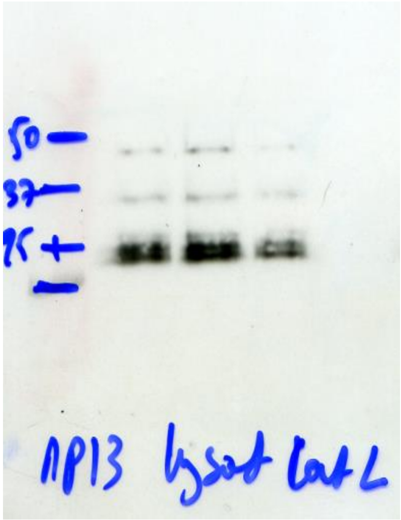

Figure 6a, CatL

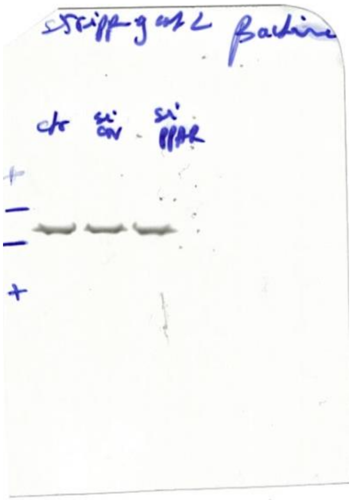

Figure 6a, β-actin

consensus PPRE

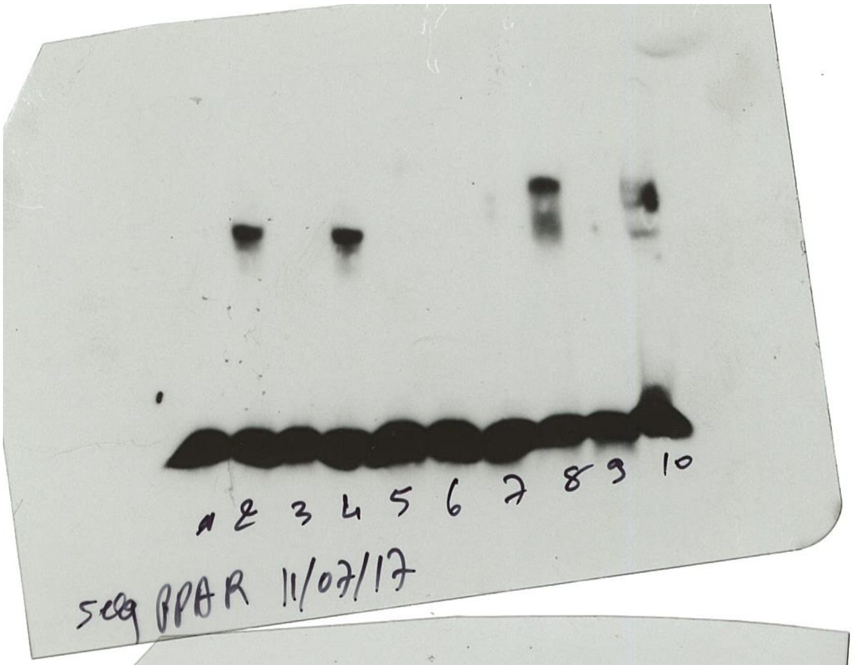

CatB PPRE

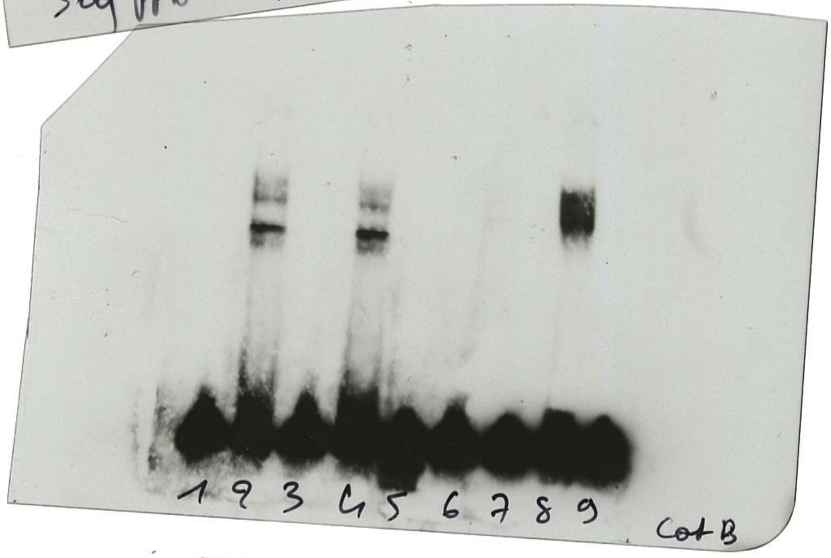

CatL PPRE

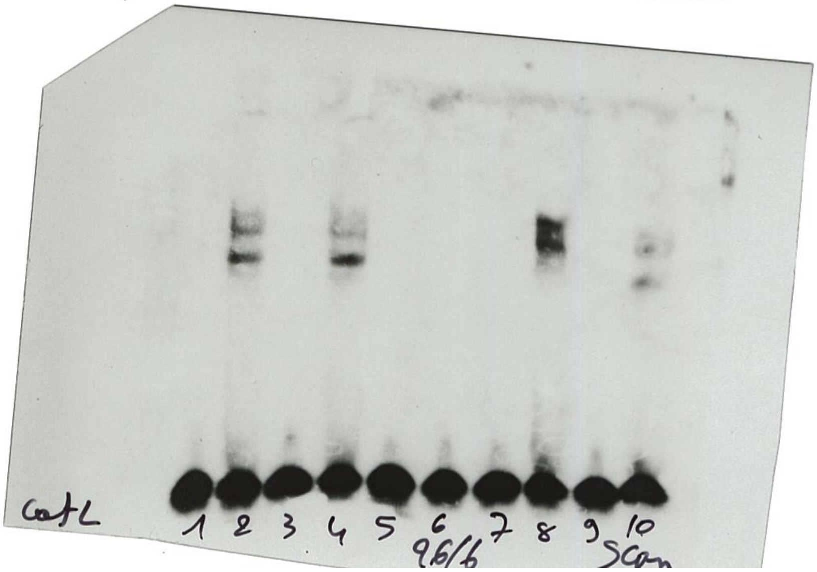

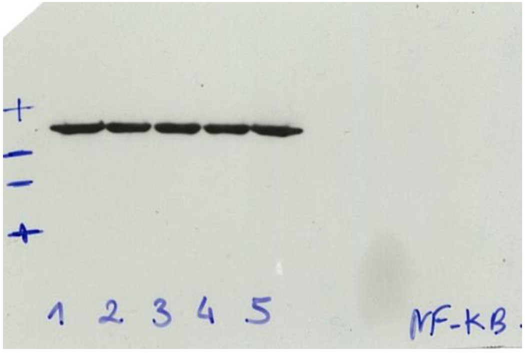

Supplementary figure 2: p65

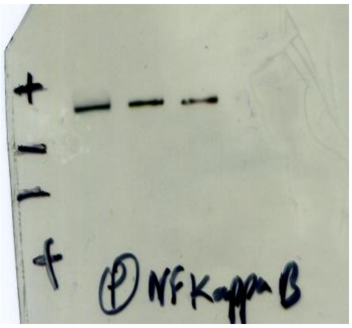

Supplementary figure 2: p-p65

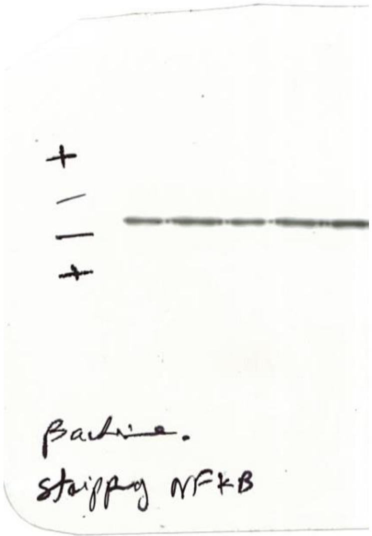

Supplementary figure 2: β-actin
